# The Italian Deaf Community at the Time of Coronavirus

**DOI:** 10.3389/fsoc.2020.612559

**Published:** 2021-01-14

**Authors:** Elena Tomasuolo, Tiziana Gulli, Virginia Volterra, Sabina Fontana

**Affiliations:** ^1^Institute of Cognitive Sciences and Technologies, National Research Council (CNR), Rome, Italy; ^2^Group for the Study and Information of the Italian Sign Language, Rome, Italy; ^3^Department of Humanities, University of Catania, Catania, Italy

**Keywords:** deaf community, COVID-19, Italian sign language, accessible information, digital education, resilience

## Abstract

The present paper will explore the impacts of the recent pandemic crisis on the Italian Deaf community, as a linguistic minority. Recent research has shown that minorities are suffering much more the effects of the pandemia because their lack of access to services and in a much wider perspective, to education and welfare. We will show that, during the COVID crisis, despite lockdown measures, various actions at the formal political level (from the Italian Deaf Association) and at the informal level (from the members of the community) promoted sign language and the Deaf community within the hearing majority. In particular, we will analyse how social networks were exploited at the grassroot level in order to promote social cohesion and share information about the coronavirus emergency and how the Deaf community shaped the interpreting services on the public media. The role of social networks, however, has gone far beyond the emergency as it has allowed deaf people to create a new virtual space where it was possible to discuss the appropriateness of various linguistic choices related to the COVID lexicon and to argue about the various interpreting services. Furthermore, in such emergency, the interpreting services were shaped following the needs expressed by the Deaf community with the results of an increased visibility of Italian sign language (LIS) and empowerment of the community. Materials spontaneously produced by members of the Deaf Italian community (conferences, debates, fairy tales, and entertainment games) were selected, as well as materials produced by LIS interpreters committed to guaranteeing access to information. By highlighting the strategies that a minority group put in place to deal with the COVID-19 emergency, we can better understand the peculiarities of that community, creating a bridge between worlds that often travel in parallel for respecting the peculiarities of each other (deaf and hearing communities).

## Introduction

From the end of February 2020, in the span of a few weeks, Italy went from the discovery of the first official COVID-19 case to a government decree that essentially prohibited all movements of people within the whole territory, and led to the closure of all non-essential business activities. Italy was the first European country to be affected by this disease. Within a very short time period, an incessant stream of deaths was announced daily, especially in the northern regions of the country. At the same time hospitals were revealed to not be equipped to deliver the type of Community-focused care needed during a pandemic. In order to avoid the virus spreading, the Italian government dealt with the COVID-19 pandemic by issuing a series of decrees that gradually increased restrictions within lockdown areas (“red zones”), which were then expanded until they ultimately applied to the entire country. On 9 March 2020, the government of Italy under Prime Minister Giuseppe Conte imposed a national quarantine in response to the growing pandemic, restricting the movement of the population except for necessity, work, and health circumstances. This included the closure of all schools, public offices, museums, galleries, theaters, stadiums, and the obligation to stay at home except for medical reasons or food shopping (from March 9th to May 18th 2020) (for more details see Regan, 2020: Italy announces lockdown as global coronavirus cases surpass 105,000”; https://hbr.org/2020/03/lessons-from-italys-response-to-coronavirus).

Recent research (Dyer, 2020; Faloppa, 2020; Pareek et al., 2020) has shown that minorities are suffering much more the effects of the pandemic because their lack of access to services and in a much wider perspective, to education and welfare. However, as we will point out in the present paper, they can also react by promoting various actions at the formal and the informal political levels in order to maintain cohesion and survive. For example, the lack of institutional sign language interpreting services, the difficulty of lipreading through the surgical masks, and the complex language register of doctors can cause misunderstandings and problems of accessibility.

In the present paper we would like to describe how a linguistic and cultural minority, the Italian Deaf signing Community, has responded to the COVID emergency. As recommended by Araabi (2020) “While we fight to mitigate the damage the crisis has wrought, we should learn lessons from the mutual solidarity and Community resilience that it has unveiled. It will ensure the world that comes after the crisis is a better one for all.”

Before exploring in detail the impacts of Coronavirus on the Signing Community, we need to briefly describe its sociological and sociolinguistic status, its linguistic repertoire and finally the relationship with the hearing majority.

## Context

The situation of deaf people in Italy is extremely diversified and stratified. Deaf people can be monolingual and use only spoken Italian, or bilingual and use Italian and Italian sign language (LIS). Such bilingualism, defined as minority language bilingualism (Grosjean, 2010), is not always balanced as often there is a strong asymmetry between the two languages (Caselli and Rinaldi, 2019; Rinaldi et al., 2019). In fact, whereas all deaf people (in Italy about 1/1000), after diagnosis of hearing loss in childhood, start speech therapy, only deaf children of deaf parents (about 5% of born deaf) are exposed to sign language since birth and acquire it spontaneously as hearing children do with speech (Rinaldi et al., 2014, 2018; Volterra, 2014). Most deaf children are born in hearing families (about 95%) and, in such cases, only rarely have access to sign language in the first years, but acquire LIS at later stages of their life. Thus, all Italian Deaf people learn and use spoken and written form of Italian and most of them also acquire and use LIS in their everyday life, although with a large individual variability in the proficiency in each language. The linguistic development of all deaf children, and in particular the learning of Italian, is always determined by some variables: the degree of hearing loss, the age of diagnosis and of use of hearing aids or cochlear implant, the kind of rehabilitation, the quantity and quality of interactions with Italian speakers or LIS signers, and finally the educational program. Italian Sign Language has not yet received full official recognition as a minority language by the Italian Government and therefore, the bilingual status of Deaf Italians also has not yet been officially recognized. Research on sign language began in Italy about 40 years ago and only since 1981 were research projects developed to describe this language, its acquisition processes as well as related historical factors. Interest in LIS also grew in the fields of rehabilitation and education, and training programs for hearing and deaf professionals started. In 1988, the European Parliament initiated the path toward recognition of all national sign languages and encouraged the publication of dictionaries and the establishment of courses and interpreting services, as well as of television programs for the Deaf. However, only in 1992 was a special Italian law adopted that, in support of students and families, offered the possibility of obtaining signing teaching assistants in schools and LIS interpreters in universities. Thanks to this law, LIS courses, bilingual educational programs, and interpreting services were often funded and supported by local governments or at the national level (Geraci, 2012; Rinaldi et al., 2014; Fontana et al., 2015). It is widely accepted that for both hearing and deaf persons the acquisition and use of minority languages are fundamental for the building of self-identity and the construction of cultural and linguistic values. However, as is often the case with minority languages, LIS is used by a restricted number of people and is often felt to be a low-status communicative code to be used only in informal settings compared to Italian, which is regarded as the majority high status language. The strong opposition toward sign language established since the Milan Congress of 1880 has had a strong and lasting influence on the hearing environment of the following generations. LIS was thus rarely considered for use within larger and public settings or by the media and this is one of the ways in which the lower status of a minority language in the Community can hamper the development of that language.

Although Italian deaf people ask for the legal recognition similarly to other minority languages (Marziale and Volterra, 2016), many of the hearing families and doctors maintain that this language is not worth learning because of the new technology advances (i.e., cochlear implants). Given this scenario, the Deaf Community has met great difficulties during the emergency for COVID-19 at various levels and contexts of everyday life. For example, mask wearing has isolated Deaf people: in fact, not only is lipreading crucial when communicating with hearing people, but also lip movements represent an important feature of sign languages as clearly explained in the following link by a Deaf member of the Community (https://www.cnrweb.tv/se-la-mascherina-maschera-le-parole-ai-sordi/). Despite such difficulties we will show how this minority managed to find new forms of organization and to suggest solutions (such as clear masks) to many of the problems that public institutions were not able to face and to solve.

## Detail to Understand Key Elements

**Figure 1:**
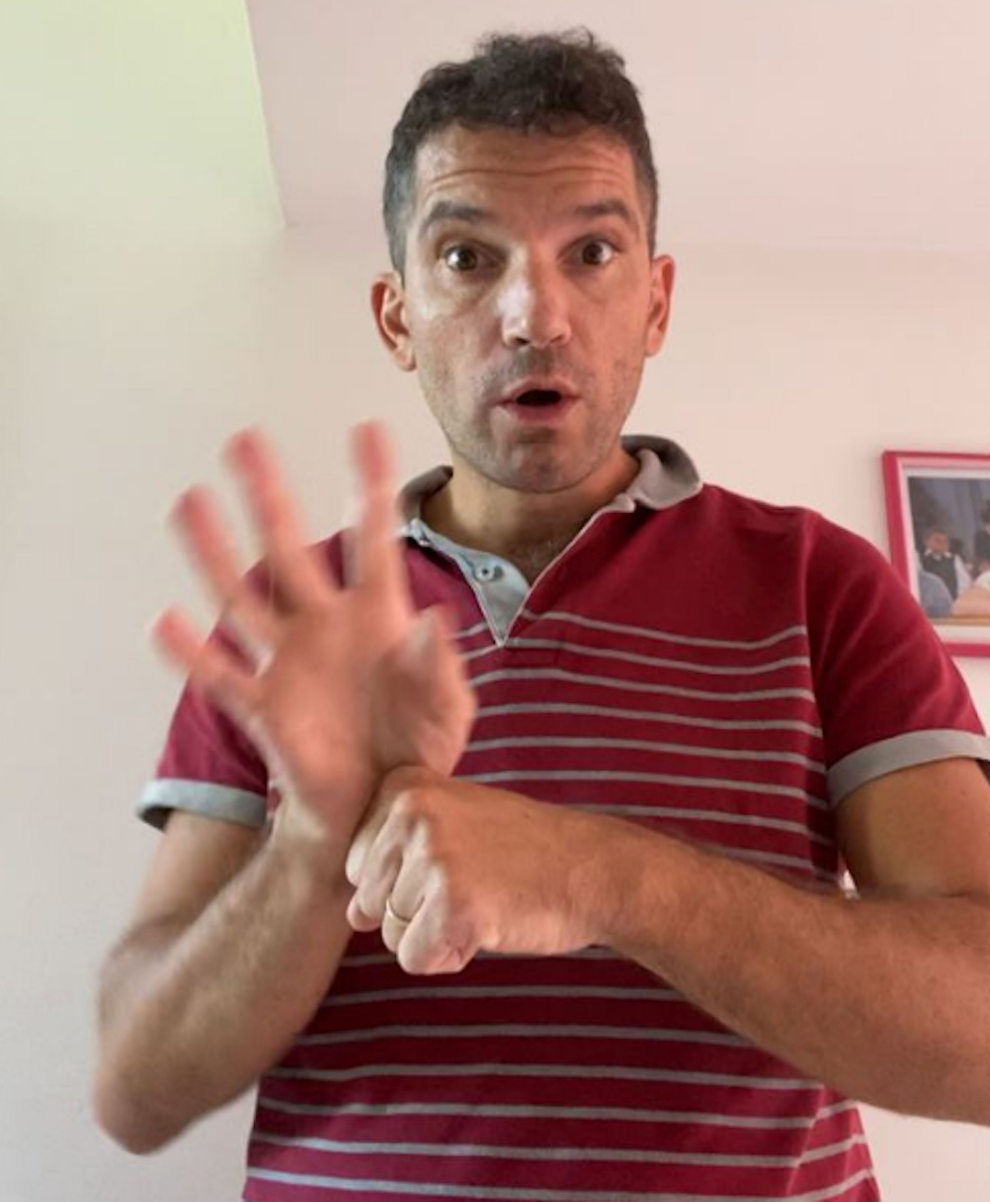
The sign for coronavirus in LIS.

In the present circumstance of the COVID-19 emergency (Figure 1), the Italian Deaf Community has assertively requested full access to information in order to participate and get their needs recognized. In many cases the Community showed to be able to find the best way to have complete information through professional sign language interpretation services.

In particular, different activities that took place in the period March-May 2020 have been described, by analyzing Facebook and the public television networks as sources. Materials spontaneously produced by members of the Deaf Italian Community (conferences, debates, fairy tales, and entertainment games) were selected, as well as materials produced by LIS interpreters committed to guaranteeing access to information (Grandi and Piovan, 2020; Gulli and Volterra, 2020; Tomasuolo and Volterra, 2020).

We will investigate these changes by taking into account the three areas: Education, Information and Cultural, and Language and Entertainment as indicators that show the level of self-organization and empowerment of the Community.

### Education

**Figure 2:**
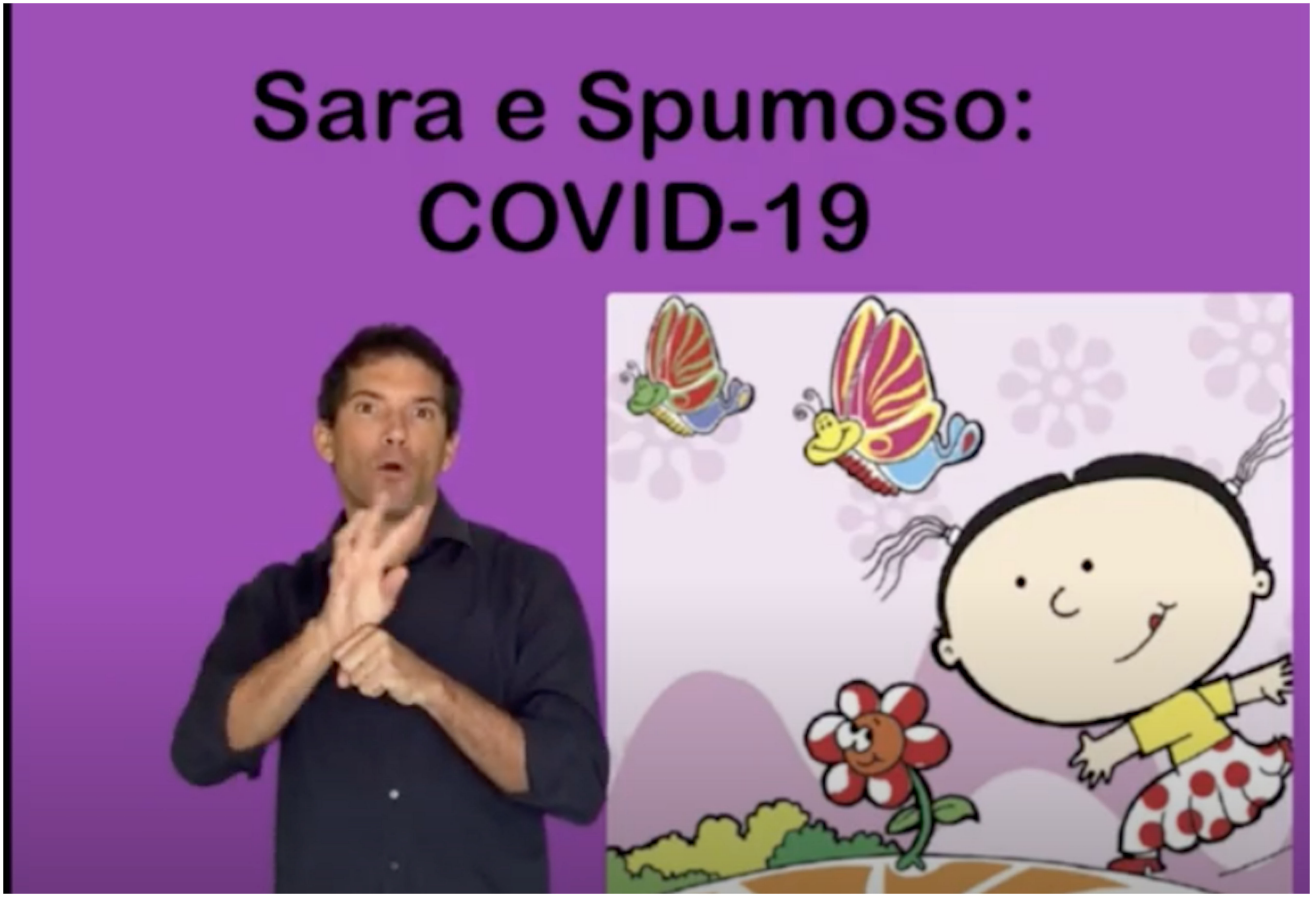
A deaf adult explains the Coronavirus emergency to young children.

During the COVID emergency, schools have been closed from the middle of February/beginning of March, (depending on regions) until the end of the school year (beginning of June). Schools began to organize “distance learning” after some puzzling moments. This reorganization presented many difficulties for all the students and in particular for the deaf ones. In the Italian public school, from 3 to 18 years, families of deaf students can request in addition to a “support teacher” (a teacher trained to assist all disabled students) the presence of a teacher assistant able to use the more appropriate linguistic modality to that student [Italian Sign Language (LIS), Signed Italian (SI), and spoken Italian in a clearer labial form]. Few public schools offer a bilingual curriculum that implies the use of Italian and LIS within a classroom attended by hearing and deaf pupils. In these schools, the teachers either use LIS, Signed Italian (SI), or there is a LIS interpreter who simultaneously translates the teacher's and pupils' messages from Italian to LIS and vice versa. In these bilingual schools, not only is there a greater possibility to receive school instruction in SL, but there is also increased opportunity for interactions in LIS or SI between deaf and hearing schoolmates as well as among hearing and deaf instructors, both within and outside of the classroom (for a description of bilingual schools in Italy, see; Teruggi, 2003; Russo Cardona and Volterra, 2007; Di Gregorio et al., 2019). At University, the students can request the presence of a sign language interpreter for a full translation of the oral lessons, and for assistance in exams and meetings with professors. In many cases during the long period of distance learning (or remote schooling) these professional services have been interrupted. Deaf students and their families actively protested for this extreme situation: for example a deaf adolescent has publicly denounced the absence of the teacher's assistance on the online platforms where her school was delivering classroom lessons (https://youmedia.fanpage.it/video/aa/Xn86ZOSwlMKQOHcg). Her protest has been accepted and a distance service of communication assistance has been activated (https://www.facebook.com/noemi.magro.1/posts/2839106389537371). The few schools practicing Italian-LIS bilingualism (https://www.istruzione.it/coronavirus/allegati/esperienze/Istituto_Comprensivo_di_Cossato_BI_rc_2.pdf) or scientific institutions such as the Institute of Cognitive Sciences and Technologies, CNR (https://www.istc.cnr.it/it/10_min_lacam_condiviso_altri_siti), delivered on the web educational materials for deaf pupils. In the early stages of the COVID emergency, public television did not produce special educational programs. Only at the end of march an inclusive cartoon for children from 2 to 6 years of age with deaf signing actors was broadcasted on Public Italian broadcasting Corporation (RAI) (https://www.raiplay.it/video/2020/03/lampadino-e-caramella-nel-magiregno-degli-zampa-s1e1-viva-il-circo-2fe0cadc-c3f2-46d6-9f85-cbbc06ed2405.html?fbclid=IwAR1xQaQs7VOrpsoDQx9iRN_uvj3j3MWGw2Rr72miiiLWOccNrWIgvA2S5bE). At the end of April RAI provided a LIS interpreter for some educational programs such as “La scuola non si ferma” (School never stops) in order to support deaf children in lockdown. In the absence of public institutions interventions, the Deaf signing Community activated the majority of distance educational initiatives during the first stages of COVID. It has organized and produced many interactive interesting activities including entertainment games quizzes, fairy tales, narrations, and theater plays. Signing adults have exhibited special creativity and unexpected skills in the production of materials which were entertaining, instructive, and useful in maintaining active interaction at a distance among different families and between children (https://www.facebook.com/StoriebambiniragazziLIS/). See in particular: https://www.facebook.com/101231048029351/videos/221939432546383; https://www.youtube.com/watch?v=PXHuANJ6Kjk. These video materials, in LIS with or without Italian subtitles have been realized in short time by private individuals (deaf parents) as well as by associations with poor technical instruments. In the future, they could become useful tools for deaf and hearing teachers of deaf pupils who often complain about the absence of educational materials specifically designed for Deaf education. Some videos were devoted to explaining the Coronavirus emergency to young children and two books have been produced through an international network by adapting the same two books to various Sign Languages and cultures (Figure 2) (https://www.youtube.com/watch?v=TXYErGdfA8Q&t=41s&pbjreload=101; https://riseebooks.wixsite.com/access/c).

An Association devoted to bilingual LIS/Italian education “Cooperativa onlus Il Treno,” in order to promote further interaction, has produced quizzes and games for young children in LIS for all signing children, deaf and hearing, who could participate and provide their responses at a distance (https://www.facebook.com/iltreno33). Furthermore, deaf signing mothers have organized special learning activities for the summer period, given that in Italy school holidays last longer than in other countries, from the middle of June to the middle of September and many deaf children do not have the possibility to interact in SL with relatives or caregivers at home (https://www.facebook.com/590173358/videos/10157319720663359). As reported by research, Sign languages usage can improve cognitive development as well as promote a deeper understanding of many school disciplines. Indeed, in the school environment, sign language guarantees full access to contents and allows students to focus on school subjects, regardless of their difficulties in reading and writing (Rinaldi et al., 2014; Di Gregorio et al., 2019).

### Information

Accessibility to information has always represented a very crucial problem for the Deaf Community. In Italy, information usually is in an oral (radio and TV) or in a written format (by newspapers, printed, and online magazines). So far LIS interpreting services have been granted only for news programs. Other programs such as movies or talk shows have been subtitled but although services have been set up involving users, the kind of involvement has been top down. In other words, users were asked what aspects of the subtitling service they would improve, and not how they would set it up.

During the lockdown, new smart ways of using visual ICT tools (Gulli and Volterra, 2020) have developed out of the strong need of the Community to be fully informed. Instead of relying on the hearing majority for interpreting and subtitling services, the Deaf Community has shaped its own accessibility, by lobbying the majority in meeting the expectations of Deaf people when setting up services. Interestingly, this has reversed the traditional roles of the relationship between majority and minority, by enlightening the new important role of minorities in building a new inclusive society. In this section, we will describe how information was made accessible and the role played by the Deaf Community in building accessibility and in guiding the local and national public institutions to improve their LIS and subtitling services.

We will analyze services from two perspectives—from the majority and from the minority—in order to show how the minority can steer services that the majority has set up. We start describing the nature of services that the majority has set up for the deaf Community and some examples of the minority reactions.

Since the 25th of February, two press conferences of the Civil Protection Direction took place daily with the presence of the LIS interpreting service. The hearing majority reacted with interest although some people misunderstood the role of the interpreter. In the online comments on the newspaper “Corriere della Sera” on the Civil Protection's bulletin, the interpreter was labeled as a “person who looks upset.” A study on similar comments and replies would be interesting. Here, we just would like to emphasize the fact that such comments show that many hearing people are not aware of LIS and of the interpreters' role.

**Figure 3:**
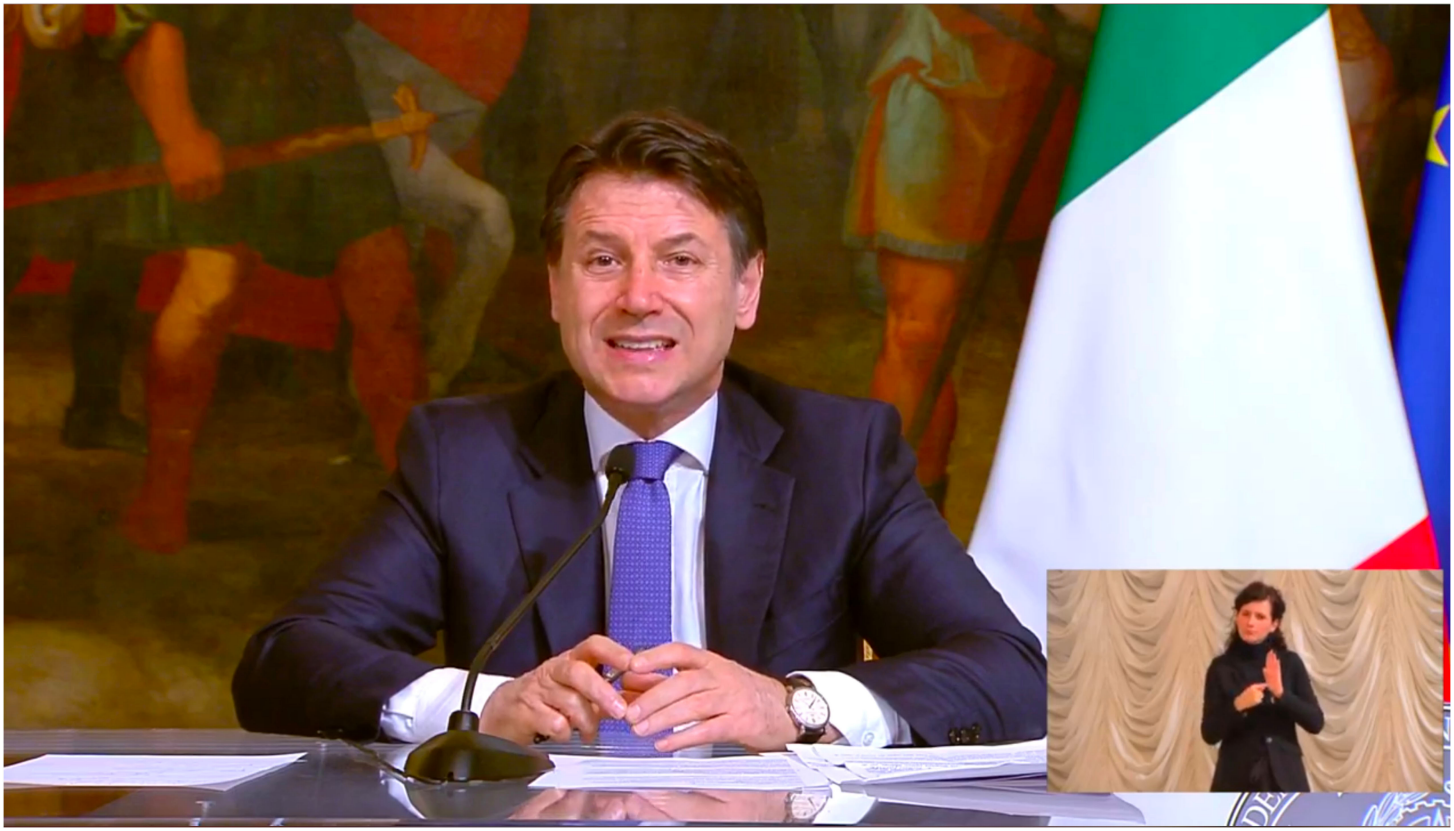
The President of the Council Conte with the interpreting service.

The Deaf Community played a crucial role in lobbying the hearing majority for improving and increasing sign language interpreting services. Here is a brief summary of the events. The Prime Minister has delivered various press releases. The first ones were subtitled and not interpreted in LIS. As a result, the 12th of March, the Italian National Deaf Association (hereafter ENS) has promoted a collective mobilization and a social protest at a national level to ask for interpreting services in LIS for the Head of the government's releases as has already happened in other countries (e.g., France, Spain). Finally, on the 21st of March 2020, the ENS announced that the battle has been won and that the Prime Minister Conte press releases will be interpreted in LIS (Figure 3). However, at about 11.30 p.m. of the day after many Deaf people were waiting for Conte's press release without knowing really in which channel would have been broadcasted. Nearly at the end of Prime Minister Conte's press release, someone noticed that the interpreter was there but that it could be visible by going on YouTube or on Facebook. The cameraman has chosen not to frame the interpreter for aesthetic reasons (!) and to foreground only the Prime Minister. Finally, on the 24th of March, the Prime Minister Conte's releases were broadcasted on the unified TV Channels with the LIS interpreter framed on the side that was partially covered by subtitles. As a consequence of the protest activated by the Deaf, the interpreter's window has been modified and moved up on the 28th of March. Furthermore, on the 25th of March, on the national TV channels the whole meeting of the Deputy's Chamber was translated in LIS. On this occasion, Conte delivered a speech on the emergency situation and was translated in LIS by two interpreters. The same happened on the 26th of March for the question time and Conte's speech on the Senate. It was the first time that such a long live broadcast has been translated in LIS. However, the President of the Republic Mattarella's releases on the national sanitary emergency were not translated, even though since 1995 the Presidents' end of the year speeches have always been translated in LIS.

Also, the World Federation of Deaf (WFD) has collaborated with international bodies to ensure information and to issue guidelines on accessibility to national governments. One example of the WFD's successful work are the WHO Disability Guidelines. Whereas, previously sign language interpretation should be provided “if possible,” now this has changed to become mandatory (https://wfdeaf.org/news/covid-19accessnow/; http://wfdeaf.org/news/access-is-not-an-option/). As stated by the General Secretary of the United Nations “It has been encouraging to see some countries providing public health announcements and information on COVID-19 with national sign language interpretation” (https://www.un.org/en/observances/sign-languages-day/message; http://webtv.un.org/watch/player/6154369287001?fbclid=IwAR1SAYwuUjo5ShTWPv2vKKjcCpQf-nxIlXbvH4IdPWMiqlMsOCyXVzc3EQk).

Many leaders indeed in Europe and in the world have activated sign language interpreting services. This has been very important both for the dissemination and visibility of sign languages and for the accessibility of information by the Deaf Community. However, not all political leaders have delivered their official speeches with a sign language (hereafter SL) interpreter: for example the USA President Donald Trump (https://www.youtube.com/watch?v=8-2wqD1LtF8) and the Prime Minister of the United Kingdom Boris Johnson, have always delivered their official speeches without any interpreter (https://www.youtube.com/watch?v=jK8vjgVlc8A; https://www.youtube.com/watch?v=J74Y-cpOdhg).

However, after a few months the official announcements from 10 Downing Street were accessible through British sign language interpreters (https://www.youtube.com/watch?v=3fbsciR8As8;)

In addition, the American National Association of the Deaf started a legal action against Trump and the Federal Court ordered that the White House must begin providing American Sign Language interpreters at all White House coronavirus briefings (https://www.nad.org/2020/09/11/judge-orders-white-house-to-provide-asl-interpreters/). Thus, since 1st October the presence of sign language interpreters at the White House should become mandatory (https://www.nbcnews.com/politics/donald-trump/judge-orders-white-house-provide-sign-language-interpreter-covid-briefings-n1240954).

As far as local and regional institutions are concerned, interpreting services were not granted even though they have recognized sign language. Generally, however, once the local Deaf Community has expressed their need, the local Institutions were ready to set up service as they need. Indeed, many Presidents of Regions and mayors were interpreted in LIS.

**Figure 4:**
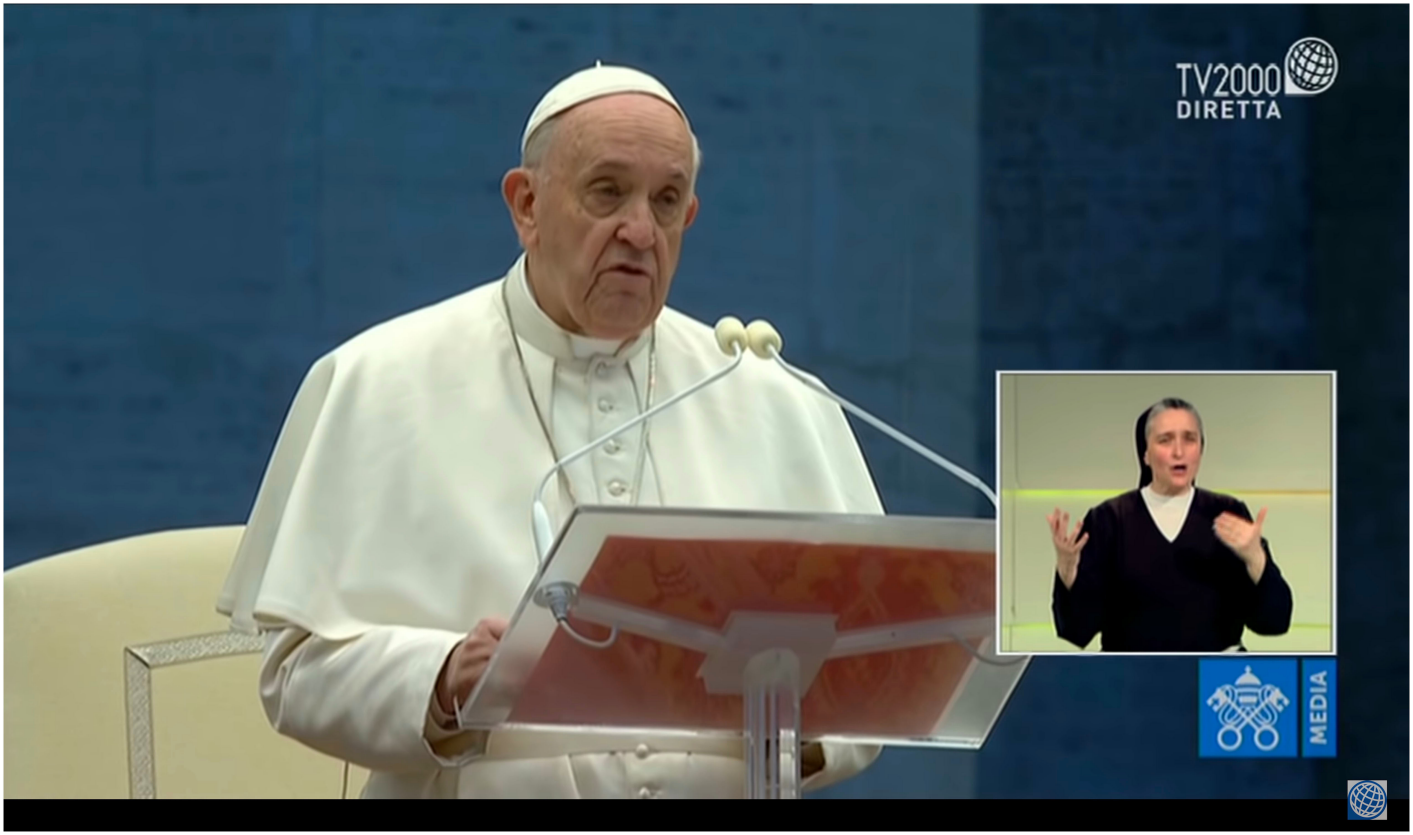
Pope Francis alone gives a homily in San Pietro square with the interpreting service.

Interestingly, also Pope Francis, on Friday the 27th of March, in the desert Saint Peter square in Vatican city, gave a homily devoted to the difficulties of the moment that was broadcasted by all TV channels with the interpreting services (Figure 4).

The Deaf Community played a very crucial role in selecting and shaping the kind of information they need access to. Many videos on the COVID-19 emergency were not accessible in sign language and this put the deaf population at risk for lack of information. In such cases, the non-profit organizations took the initiative to translate such videos and grant to the Deaf Community the basic information. For example the Cooperative “Segni di Integrazione Piemonte - Paolo Basso” in collaboration with ENS worked to translate the most important information on Facebook.

The President of the Italian National Deaf Association (ENS), has delivered a long interview to a Deaf interviewer on the deaf people accessibility on the emergency period for COVID-19 (https://www.facebook.com/Micel70/videos/10158160381593256; https://ens.it/coronavirus).

In this interview, he highlighted the strong difficulties that deaf people met during lockdown and the crucial role of the Deaf Association in lobbying the government in order to tailor services to the need of all Deaf people, especially old Deaf people and children.

Extended interpreting services for official releases have established a new role for LIS in the Hearing and Deaf Communities. LIS shifted from a private language used only in familiar informal settings to a public language with new functional dimensions (Cuxac and Antinoro Pizzuto, 2010; Fontana, 2020). It is an important revolution not only at the linguistic but also at the social level, because social networks can ensure the participation of all Deaf people through generations to events that were set up only for the mainstream, thus promoting shared experiences for the majority and the Deaf minority. Furthermore, in such emergency, the interpreting services were shaped following the needs expressed by the Deaf Community with the results of an increased visibility of Italian sign language and empowerment of the Community. To summarize: the Deaf Community played a very important role not only in increasing the interpreting services but also in tailoring them to the needs of the various members of the Community, from the younger to the older generation. Thanks to their intervention, the visibility of interpreter was improved in all the news and release programs.

In the following sections we will describe the various initiatives at the grassroot level that promoted social cohesion and sharing information about the coronavirus emergency. We will point out how social networks allowed deaf people to create a new virtual space where it was possible to discuss many topics.

### Cultural, Language, and Entertainment Activities

At the beginning of the lockdown, various actions have been promoted to maintain social cohesion and encourage Deaf Community members to face isolation and everyday difficulties. One example is the flash mob campaign “#I stay at home” promoted by a Deaf activist who has created a Facebook group called “Passa segni LIS positivi” (Spread LIS positive signs) (https://www.facebook.com/rosella.ottolini/videos/10222441255123958/). In few days hundreds of Deaf Community members have repeated the same little song with few modifications: some families, including seniors and children, have played the same content all together.

Some well-known Italian actors and football players have participated to the campaign by learning LIS signs and repeating the same slogan “#I stay at home” (https://www.youtube.com/watch?v=sH-NpMYmOJA). Other European countries, as France and the Netherlands have imitated the same text with few modifications in their sign languages (https://www.facebook.com/groups/134664384637913/permalink/140217037415981/; https://www.facebook.com/hilde.verhelst/videos/10156789465957274/).

These examples clearly show how relationships and human solidarity have immediately established among Deaf Communities not only from Europe but also from other non-European countries. In the following link a Deaf Chinese boy who lived in Rome, explains the situation reassuring that Chinese experienced doctors were going to help Italian colleagues (https://www.facebook.com/morelli.qgio/videos/2837779289592152/).

Many members of the Italian Deaf Community have spread information and promoted debates in various cultural areas: art history, philosophy, psychology, linguistics.

For example, a Deaf art historian has described in detail a famous painting by the Dutch painter Pieter Bruegel the Elder, entitled “The Triumph of Death” (1562) and kept in the Prado Museum (Spain). The painting depicts a plague epidemic similar to COVID-19. The LIS description underlines many similarities between the past and the present situation: as in ancient times, the Jews were accused of having spread the plague, nowadays the Chinese population has been blamed for the epidemic (https://www.facebook.com/violante.nonno/posts/10219470242579406).

Some interventions concerned philosophical and psychological topics. In one video a Deaf mother and her daughter have discussed in the form of a dialogue about the survival instinct (https://www.facebook.com/enza.giuranna/posts/10215592483714388).

Another Deaf Community member has signed the history of Anne Frank, the Jewish girl forced to remain for more than 2 years in a very restricted space in Amsterdam to escape from the Nazis. In this description many differences between her situation, who had only the diary as resource and the current lockdown context suffered by Italian people who had the support of many technological aids were paralleled.

A Deaf psychotherapist has delivered some videos concerning the psycho-physical wellness and the best ways to manage emotions in the period of COVID-19 emergency (https://www.youtube.com/user/mmottinelli/videos).

Some activities have been expressively dedicated to the Coronavirus (Visual Vernacular on Coronavirus performed by an Italian deaf - https://www.youtube.com/watch?v=wMBhzVjfaDo), whereas others were devoted to cheer up and maintain Deaf and as well as hearing people in good mood. A hearing singer has adapted the famous Neapolitan song “Tu vuò fa l'americano” (You want to be American) to the COVID situation with the new title “Tu vuò fa la quarantena” (You want to be in quarantine) and a CODA interpreter and music expert has adapted the performance in LIS (https://www.facebook.com/mauroiandololisperformer/posts/2826228984164812). And blessed be humor, the best way to withstand all this. When we are able to laugh at the coronavirus, we are actually saying that it has not yet driven us into total paralysis (Grossman, 2020).

The linguistic choices made by the signers are one of the most interesting aspects of these social events.

The COVID emergency has constrained all languages and cultures to introduce in everyday communication new concepts and technical labels. In the case of Italian sign language, some signs have extended their meaning and some terms have been borrowed by other sign languages and adapted to LIS. Gianfreda et al. (2021) have described in detail some LIS signs used to describe the coronavirus and the quarantine experience. In some cases the signing Deaf Community members have started to discuss some linguistic choices and variations in spontaneous expressions or translations from Italian. For example, one of the debates concerned the concept of positive, which can be used to describe very different contexts: a positive result of a test to check the presence of the virus or an ongoing pregnancy. Many people have participated in the linguistic debate at a distance explaining that to distinguish the different meanings and to avoid misunderstandings, a positive or a negative facial expression should accompany the manual sign or further signs have to be added to clarify the content (https://www.facebook.com/rosella.ottolini/videos/10222286641378711/; https://www.youtube.com/watch?v=fkbQyzeb2ao).

## Discussion

The last pandemic crisis, known as *Spanish flu*, happened in 1919, about a century ago, but we do not have many memories, data, nor bibliographical references on the events concerning the Deaf people. Unfortunately, then, we lost much important data about the ways the Deaf Community coped with that event. Conversely, the Coronavirus lessons should remain when the crisis comes to an end (Araabi, 2020). In the present paper we have referred to the Italian situation but it is possible that the same happened also for other Deaf Communities around the world and we hope that such a survey will start soon in other countries. In our case, we shall never forget how the Italian signing Community has self-organized and gained in empowerment and resilience. The numerous videos online should be saved and analyzed to be re-used in the future by teachers, parents, or professionals working in the field of deafness. All these videos could be useful for teaching to deaf children, for training school support teachers and other professionals or for interpreters who have to make information accessible for the Deaf Community.

We need to learn from Deaf people, taking advantage from what the Community has produced for itself and for its children when working out solutions or paths not only for them but also for the hearing majority. For example, the use of the clear masks can improve lipreading in both deaf and hearing people or enhanced visual learning could be useful for deaf as well as hearing students.

Within the Deaf Community, leading members shared the responsibility of promoting the access to information for everybody, signing families felt the importance of sharing signed videos with other hearing non-signing families with deaf children with a strong impact on the hearing majority attitudes toward the signing Community. In the lack of institutional SL interpreting services, also the hearing Community set up informal ways to communicate with deaf: for example in an Italian hospital the staff communicated with a COVID deaf patient by writing messages on paper.

In all these events, the Community used its own language, showing its value and effectiveness.

All these actions should be made more visible and shared because, being built by the Deaf Community, they highlight the pathways toward a more inclusive society.

The Coronavirus showed the fact that Community-led responses are much more effective compared to those coming from centralized governmental actions that very often reflect and reinforce existing inequities. Also, it showed how social networks can turn into strategies to empower, to inform and keep people together. Social networks also represent the new challenge for the future: they are not only the “place” where these incredible experiences will be archived but also the starting point for building a new sense of Community.

## Data Availability Statement

The original contributions presented in the study are included in the article/supplementary materials, further inquiries can be directed to the corresponding author/s.

## Author Contributions

This article was co-written by all four authors. ET wrote in particular the Introduction, Detail to understand key elements and the paragraph about Education. TG the paragraph about Cultural, Language, and Entertainment activities. VV the Discussion section. SF the section about Context and the paragraph about Information. The Manuscript contribution to the field was conceived and written by all the authors together.

## Conflict of Interest

The authors declare that the research was conducted in the absence of any commercial or financial relationships that could be construed as a potential conflict of interest.
